# Two possible *in vitro* alternatives to evaluate the effect of gastric acid on resin-based composites

**DOI:** 10.4317/jced.59782

**Published:** 2022-09-01

**Authors:** Alexandra Gil-Pozo, Priscilla Medina-Sotomayor

**Affiliations:** 1 Universidad de Cuenca, Facultad de Odontología, Cuenca, Ecuador; 2 Universidad Católica de Cuenca, Carrera de Odontología, Campus Universitario Azogues, Ecuador

## Abstract

**Background:**

Objective: To compare two *in-vitro* protocols to study the effect of simulated gastric acid on the mechanical properties of resins based composites(RBCs).

**Material and Methods:**

Three RBC FILTEK Supreme XTE (FS), BRILLIANT EverGlow (BE), GrandioSo (GS) were used. They were randomly divided into a control group (CG) and two groups exposed to simulated gastric acid: a 6-month daily protocol (DG) and an accelerated 90-min protocol (AG). Vickers microhardness (VH) and flexural strength were evaluated at baseline and six months. Statistical analysis was performed using repeated measures ANOVA tests for VH and three-way for flexural strength data (α=0.05).

**Results:**

Daily exposure in the CG and DG groups caused a reduction in VH values and flexural strength (*p*<0.05). The majority of values in the AG remained stable, after an exposure of 90 min; FS (*p*=0.118) and GS (*p*=0.729) in VH and FS (*p*=0.377), BE (*p*=0.692) and GS (p=0.672) in flexural strength.

**Conclusions:**

Daily exposure during 6 months caused significant changes in the VH values and flexural strength of the RBCs. The acid-accelerated protocol did not cause the same magnitude of change in VH values and flexural strength seen at six months of daily exposure.

** Key words:**Gastric acid, hardness, composite resins, flexural strength, dental materials.

## Introduction

During life, teeth are exposed to a series of physical and chemical attacks that act together and contribute to the wear of the dental structure. Dental erosion is one of the main causes of dental wear. The low pH and erosive capacity of gastric acid (intrinsic) are significantly greater than those of acids from diet or medications (extrinsic), so the level of destruction is usually more severe (1,2). According to recent epidemiological data, dental erosion in adult patients with gastroesophageal reflux disease (GERD) has a prevalence between 24% and 32.5%. (3)

Today, given the awareness of the need to preserve the greatest amount of tooth structure, even more so in those dentitions that already show signs of wear, it is crucial to select the appropriate restorative material. (4) Due to improvements in adhesive materials (5-8), it has become possible to rehabilitate eroded teeth in a less invasive way using direct restorations with RBCs (9). However, these materials inevitably undergo aging, which is influenced by the dynamic and complex environment of the oral cavity (10,11). The degradation of RBCs is a somewhat complex phenomenon that involves mechanisms such as the hydrolysis of both the polymeric network and the silane bonds between the fillers and the matrix, causing the plasticization of the polymeric matrix and therefore the elution of filler particles as well as components that have not reacted (12). This phenomenon results in a decrease in some physical and mechanical properties of the material, such as hardness and flexural strength (13,14). RBCs are hydrophobic but contain hydrophilic monomers (12). The presence of these monomers in different proportions would explain why RBCs cannot be considered inert materials in aqueous media (15) and exhibit even worse properties in circumstances with low pH, as in the case of gastric acid pH 1.5 - 3.0 (15,16).

Although the best environment to evaluate the performance of a material will always be the oral cavity, *in vitro* tests are useful and are widely used to observe the behavior of materials under different circumstances and provide valuable information for future clinical and research (17). Unfortunately, the heterogeneity of experimental designs with respect to intrinsic erosion (2,13,18-22) makes it difficult to compare results and safely extrapolate to the clinical environment (23,24).

Given the lack of consistent evidence in studies analyzing erosion and to observe whether acidic pH values of gastric origin alter the properties of RBCs, the present study aims to evaluate the possible effect of acidic pH values on the mechanical properties, i.e., Vickers hardness (VH) and flexural strength, of RBCs exposed to two types of protocols (daily or accelerated) that simulate endogenous erosion for six months.

## Material and Methods

The present study examined three RBCs: FILTEK Supreme XTE (FS; 3M ESPE - St Paul, Minnesota, USA), BRILLIANT EverGlow (BE; Coltene/Whaledent AG - Altstatten, Switzerland) and GrandioSo (GS; Voco GmbH - Cuxhaven, Germany) (Table 1).

**Table 1:**
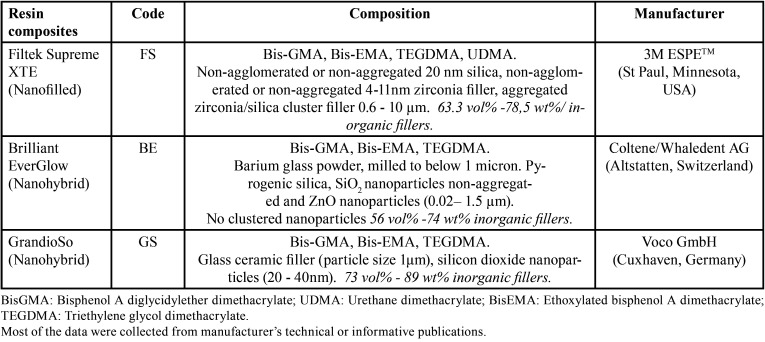
Materials used and their composition.

The specimens for VH analysis were made in a cylindrical stainless steel mold (Ø 10 x 1.5 ± 0.05 mm (Smile Line USA Inc., Colorado, USA). For the flexural strength tests, a custom mold was used, and samples of 12 x 2 x 2 ± 0.01 mm were obtained (25). The RBCs were packed inside each mold and polymerized for 40 seconds on each side (800 mW/cm2) with a polymerization lamp (Valo, Ultradent Products Inc., South Jordan, UT, USA) in a polymerization chamber (VISIO BETA vario, 3M/ESPE, Seefeld, Germany) for 7 min. All samples were polished under cooling with abrasive discs of silicon carbide P500, P1200, P2400 and P4000 (LaboPol-1, Struers, Willich, Germany). After polishing, the thickness of each specimen was confirmed with a digital caliper (Coolant Proof Micrometer IP65, Mitutoyo Corporation, Kanagawa, Japan), and the specimen was radiographically examined (RXDC Extend, MyRay, Bicocca, Italy) to rule out the presence of internal defects introduced during preparation. Finally, the specimens were cleaned in an ultrasonicator for 10 min.

Table 2 explains the two protocols performed to simulate endogenous erosion for six months. A total of 210 samples per RBC were used and were assigned by simple random sampling (www.random.org) to seven groups (n = 30). Three groups were used for VH analysis: a control group (CG, distilled water), a daily protocol group (DG, six months with simulated gastric acid) and an accelerated protocol group (AG, 90 min with simulated gastric acid). Each specimen was measured at two times, T1 (baseline) and T2 (six months). Four groups were set for flexural strength analyses: the initial group (BG), a control group (CG, six months with distilled water), a daily protocol group (DG, six months with simulated gastric acid) and an accelerated protocol group (AG, 90 min with simulated gastric acid).

**Table 2:**
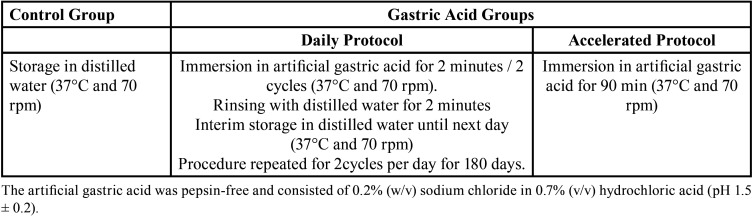
Specimen treatment protocol.

Microhardness (n = 30/group) was evaluated in a Vickers diamond microindenter (HMV-2 microhardness tester, Shimadzu Corp., Kyoto, Japan). A load of 980.7 mN for 15 s was used as the measurement parameter stipulated by ISO 6507-1: 2018. (26) The mean value of VH for each specimen was calculated as the average of five measurements at least 1 mm apart. At the beginning, the VH of all the specimens was measured (T1), and after six months or 90 min depending on the protocol (T2), the same samples were used to provide a control over time.

Flexural strength (n = 30/group) was determined with a three-point universal test kit (Model 4502, Instron Corp., Canton, Mass., USA). A 5 kN load cell with a crosshead speed of 0.75 ± 0.25 mm/min was used, according to the ISO 4049/2009 standard (26). The distance between the supports (span distance) was set at 10 mm for flexural strength analysis (25). From the maximum recorded load, the uniaxial flexural strength was calculated as α=3Pl/(2bh2), where *P* is the maximum load exerted on the sample (in Newtons), l is the distance between the supports (10 mm), b is the sample width (mm), and h is the sample height. Before testing, the initial specimens were submerged in water for 24 hours as indicated by ISO 4049/2009.

-Statistical analysis

G*Power software by the University of Düsseldorf was used to calculate the sample size for microhardness analysis. The study by Backer *et al*. (16) was considered as a reference for the estimation of standard deviation, and a power of 90% and a confidence level of 95% were established. For the flexural strength tests, a sample size of 30 specimens was selected, as previously published (27).

Statistical analysis was performed with SPSS software (Version 20, SPSS Inc., Chicago, IL, USA). Using the Kolmogorov–Smirnov test, it was found that the distributions of VH and flexural strength values were adjusted to normal, so a parametric approach was used. Repeated-measures analysis of variance (ANOVA) with interactions between RBC and group was performed for the comparison of VH results. For the study of flexural strength (independent samples), three-way multifactorial ANOVA was performed, with RBC, group and time as factors. For multiple comparisons, the Bonferroni test was applied to adequately control for type I statistical error. The reference significance level was 5% (α = 0.05).

## Results

**Figure 1:**
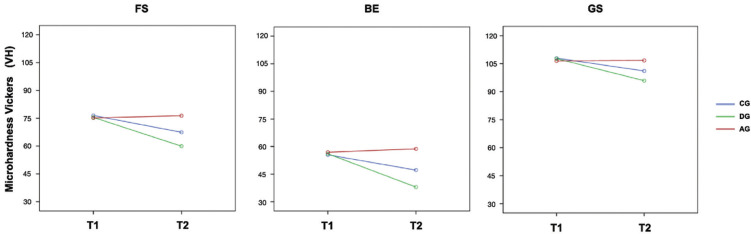
Line graphs for the mean values of VH, more intuitive to appreciate the interaction.

Table 3 shows the mean (SD) VH values under the different study conditions. Figure 1 shows the triple interaction RBC - group - time (*p* <0.05). The groups generated different levels of hardness, and the difference depended on the type of RBC and the time of evaluation. After six months, FS resulted in a reduction in mean VH of 20.5% in the DG group, while a change of 11.8% was observed in the CG group, and VH was practically stable in the AG group (+ 1.6%). With BE, the corresponding percent losses were 32% and 14.6%, and a slight gain was also observed for the AG group (+ 3.4%). Finally, with GS, losses of 10.9% and 6.3% and stability for the FS specimens (+ 0.3%) were observed. At six months, all specimens of the CG and DG groups showed significant changes in VH between one another and with respect to the initial values, while the variation in VH in the AG group was not significant for FS (*p* = 0.118) or GS (*p* = 0.729).

**Table 3:**
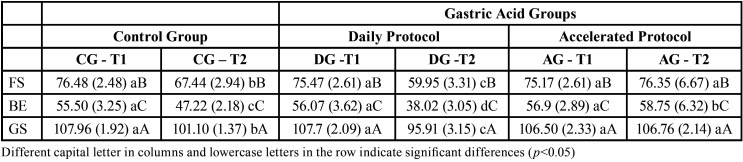
Microhardness in Vickers (VH): Mean (SD) for tested RBCs according to period of exposure to the different media.

Table 4 shows the mean (SD) values for flexural strength for all the RBCs studied. The general levels of resistance became more homogeneous between the different RBCs. Specifically, FS and GS exhibited very similar levels but the values were higher than those of BE (*p* <0.05).

**Table 4:**
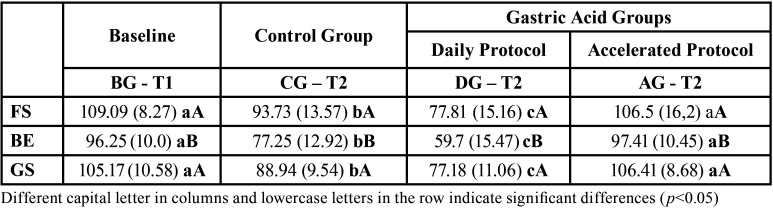
Mean (SD) values for flexural strength (MPa) according to period of exposure to the different media.

**Figure 2:**
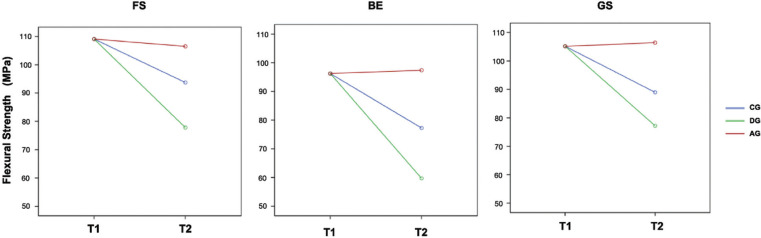
Line graphs for the mean values of flexural strength, more intuitive to appreciate the interaction.

Figure 2 shows that the difference in resistance over time depended on the protocol used (double interaction time - group with *p* <0.001) and that the pattern was similar for the three RBCs (triple interaction with *p* = 0.440.).

At six months, a significant loss of flexural strength was observed in the CG and DG groups, with variations between the means of 28.7% and 14.1% for FS, 38.0% and 19.7% for BE, and 26.6% and 15.5% for GS. In the AG group, there was no evidence of significant changes in FS (*p* = 0.377), BE (*p* = 0.692) or GS (*p* = 0.672) after 90 min.

## Discussion

The objective of the present study was to evaluate the mechanical properties of three RBCs between two protocols (daily or accelerated) that simulate endogenous erosion for six months, as well as a control group with distilled water. Daily exposure is essential to develop changes in the mechanical properties of RBCs, regardless of the medium to which they are exposed, although the simulated gastric acid drove the changes in VH and flexural strength of the RBCs.

The aging of RBCs in the oral cavity is unavoidable due to intermittent or continuous exposure to chemical agents that cause their degradation (10,28). However, the immersion time is an important factor for the deterioration of the properties of RBCs. The mechanical properties decrease continuously until the polymer network stabilizes upon saturation; normally, RBCs reach saturation in 7-60 days (28). Degradation, which consists of hydrolysis of the polymeric networks, such as the silanol bonds between the filler and matrix or the cracking of the polymeric matrix, can be absent or continue without significant changes until saturation is reached (13). This phenomenon would explain why hardly any changes were found in the mechanical properties of the RBCs when using the accelerated protocol. Despite the low pH of the simulated gastric acid, it may have been that 90 min of immersion, although designed to simulate six months of erosion, was not enough to cause the hydrolytic process, which is reflected by the significant reductions in the mechanical properties of the RBCs. In addition, the RBCs are composed of polymeric networks that are highly cross-linked by covalent bonds, which slows the entry of the solvent into the matrix (15,22).

Recently, the erosive stability of RBCs has been increasingly studied following accelerated protocols (13,16) consisting of continuous exposure durations between 12 hours and five months with different concentrations of HCl, with the objective of predicting the degradation of RBCs over several years in patients with erosion. However, no significant decreases in VH (16) or flexural strength (13) have been reported with respect to the initial values. In contrast, in the present study, BE and GS showed a slight increase in flexural strength after continuous exposure for 90 min. Egilmez *et al*. explained that the initial presence of solvent in the matrix will only blunt the tips of cracks present in the interior, reducing stress concentration and propagation. Further prolonging the exposure time is all that is required to observe the true effect of simulated gastric acid on RBCs (29).

Currently, there is no clear consensus in the literature on the best method of simulating erosion and the equivalent exposure time needed to replicate an *in vitro* model, but there are certain important variables to consider when studying erosion (23,24). Although acidic pH has been reported to have an effect on the mechanical properties of RBCs after continuous exposure for two or five weeks (13), a clinically appropriate range should not be exceeded; that is, several short bursts of no more than a few minutes alternating with cycles of saliva or distilled water are preferable, since such conditions better reflect erosive attack in the oral cavity. In this study, two cycles were used based on an average of two daily meals since reflux or vomiting events are postprandial (28), and a period of two minutes, a time also considered by another author, (24) was selected because the pH of oral fluids regains neutrality one to three minutes after the presence of acid.

Six months of storage with daily erosive exposure allowed the entry of the solvent into the polymeric networks until saturation was reached, and thus, the mechanical properties could be correctly evaluated.

The RBCs showed significant reductions in VH and flexural strength after six months of exposure, as also observed in other studies of erosive resistance (13,20,22). Hygroscopic and hydrolytic effects in the networks of dental polymers depend on the polarity and typology of the polymeric matrix, the system of the inorganic fillers and the solvent (12). The main effect of the solvent is to reduce the interactions between the chains of the polymeric network, causing the plasticization of the matrix and affecting the integrity and stability of the properties of RBCs (12,14). The three RBCs studied had almost the same polymeric matrix composition; however, their inorganic compositions were different. Therefore, the differences in VH and flexural strength between the RBCs could be explained by the size, shape and quantity of filler particles present in the materials (6,4,28).

Among the RBCs studied, BE showed the highest percent reduction in the mechanical properties tested. Two possible reasons could be argued: on the one hand, the low percentage of filling in volume of BE (56 vol% -74 weight%) compared to FS (63.3% vol% - 78.5 weight%) and GS (73 vol% - 89 weight%) could be responsible. RBCs with the highest filler content and a small particle size positively influence the properties of the material. The incorporation of a greater amount of filler in the polymer matrix can reduce the free volume available for water absorption, in addition to allowing the filler to act as a protective agent of the matrix, which is vulnerable to hydrolysis (6). On the other hand, BE is composed of barium glass powder, which is considered a radiopaque glass. This type of filler has exhibited greater dissolution in water and saline solutions than have fillers containing silica or pure quartz, which are inert in water (28). The absence of radiopaque glass, as well as the higher percentage of filling and therefore lower percentage of matrix, would explain the greater erosive resistance of GS than the other RBCs tested, as seen in a similar study (11).

Exposure to simulated gastric acid caused a marked deterioration in both VH and flexural resistance with respect to the initial values and the control group, consistent with previous studies (20,22). Chemically, gastric acid acts as a powerful plasticizer that accelerates the sorption and solubility processes of RBCs, enabling the erosion of the material (20). Acids provide a sufficient concentration of protonated protons (H+) that catalyze the hydrolysis of ester groups in the matrix. The products resulting from hydrolysis, such as alcohols and carboxylic molecules, accelerate degradation by further reducing the pH within the matrix (12). Acids can also cause erosion on fill surfaces, contributing to the leaching of the fill and leaving a rougher surface (20,22). Although simulated gastric acid is a significant source of deterioration, time is still an essential factor for observing an effect. This would explain why in previous studies, (20,22) exposure to gastric acid for a short time did not cause significant changes in VH.

A limitation of this study is it was a short-term *in vitro* study with only three RBCs. It would be highly valuable to validate a protocol to study *in vitro* erosion of endogenous origin, as well as to study the erosive stability of the properties of materials with different compositions.

Reductions in microhardness and flexural strength after six months were evident. The changes were more pronounced in RBCs exposed to simulated gastric acid, with acidic pH accelerating the reduction.

Time is a fundamental factor affecting the mechanical properties of RBCs, regardless of the medium to which they are exposed. The 90 min duration of the accelerated protocol was not enough to cause the same magnitude of changes in VH and flexural strength seen with six months of daily exposure.
